# Strain Differences in Fitness of *Escherichia coli* O157:H7 to Resist Protozoan Predation and Survival in Soil

**DOI:** 10.1371/journal.pone.0102412

**Published:** 2014-07-14

**Authors:** Subbarao V. Ravva, Chester Z. Sarreal, Robert E. Mandrell

**Affiliations:** Produce Safety and Microbiology Research Unit, U.S. Department of Agriculture, Agriculture Research Service, Western Regional Research Center, Albany, California, United States of America; State Key Laboratory of Pathogen and Biosecurity, Beijing Institute of Microbiology and Epidemiology, China

## Abstract

*Escherichia coli* O157:H7 (EcO157) associated with the 2006 spinach outbreak appears to have persisted as the organism was isolated, three months after the outbreak, from environmental samples in the produce production areas of the central coast of California. Survival in harsh environments may be linked to the inherent fitness characteristics of EcO157. This study evaluated the comparative fitness of outbreak-related clinical and environmental strains to resist protozoan predation and survive in soil from a spinach field in the general vicinity of isolation of strains genetically indistinguishable from the 2006 outbreak strains. Environmental strains from soil and feral pig feces survived longer (11 to 35 days for 90% decreases, D-value) with *Vorticella microstoma* and *Colpoda aspera*, isolated previously from dairy wastewater; these D-values correlated (*P*<0.05) negatively with protozoan growth. Similarly, strains from cow feces, feral pig feces, and bagged spinach survived significantly longer in soil compared to clinical isolates indistinguishable by 11-loci multi-locus variable-number tandem-repeat analysis. The curli-positive (C^+^) phenotype, a fitness trait linked with attachment in ruminant and human gut, decreased after exposure to protozoa, and in soils only C^−^ cells remained after 7 days. The C^+^ phenotype correlated negatively with D-values of EcO157 exposed to soil (*r*_s_ = −0.683; *P* = 0.036), *Vorticella* (*r*_s_ = −0.465; *P* = 0.05) or *Colpoda* (*r*_s_ = −0.750; *P* = 0.0001). In contrast, protozoan growth correlated positively with C^+^ phenotype (*Vorticella*, *r*_s_ = 0.730, *P* = 0.0004; *Colpoda*, *r*_s_ = 0.625, *P* = 0.006) suggesting a preference for consumption of C^+^ cells, although they grew on C^−^ strains also. We speculate that the C^−^ phenotype is a selective trait for survival and possibly transport of the pathogen in soil and water environments.

## Introduction

*Escherichia coli* O157:H7 (EcO157) responsible for over 200 infections in a large multi-state outbreak [1] related to consumption of spinach was traced back to produce grown in central California coast [2]. Major multi-country outbreaks associated with produce indicate that pre-harvest contamination has occurred often, so it is critical to identify sources of pathogens and interventions for minimizing them [1]. Indeed, EcO157 isolates that are genetically indistinguishable from the 2006 spinach outbreak strain were isolated from feral swine, cattle, and water samples near spinach fields from the central coast of California [2]. These results indicate wide-spread occurrence of this pathogen, and minimizing pre-harvest contamination will require an understanding of the biological and environmental factors that regulate its proliferation and transport from animal reservoirs to watersheds and produce grown in proximity.

There are numerous habitats in the vicinity of produce production, each of which may affect survival of EcO157 differently. Although most EcO157 strains decrease rapidly in the soil and manure environments [3], [4], and to lesser extent in water [5], [6], a small proportion of cells remain viable for extended periods. Therefore, it is probable that most EcO157 cells within a population exposed to stressful environments outside the animal host fail to survive [7]. Nevertheless, survival of some cells in water and soil results in transport of pathogen by irrigation and wind, possibly leading to produce contamination.

A mechanism for increased survival of *E. coli* and other pathogenic bacteria is through proliferation within vacuoles [8], [9] of protozoa in the environment. Passage through protozoa provides the pathogenic bacteria a survival advantage, possibly by aiding in their persistence in inhospitable aquatic environments such as chlorinated waters [8], [10] and may also increase the virulence of human pathogens [11], [12].

Direct evidence was reported for sequestration of EcO157 in vacuoles of protozoa isolated from store-bought spinach and lettuce [13]. Conversely, we reported that predation [14], [15] was associated with rapid declines of EcO157 in dairy wastewater [16] and inhibition of predation resulted in enhanced growth of EcO157 [17]. We isolated *Colpoda aspera, Vorticella microstoma* and *Platyophrya* sp. from dairy wastewater that consumed EcO157 in preference to native bacteria [14]. Although it appears that some EcO157 strains escape predation selectively due to heightened natural anti-predatory defenses, it is unclear if such mechanisms are strain specific or sub-populations and/or phenotypes exist that are resistant to predation, thus extending survival.

Survival of specific strains of EcO157 in sufficient numbers to cause infection is associated with their intrinsic fitness traits and genetic makeup [18]. Often this involves the formation of biofilms, facilitated by the production of adhesins and polysaccharides [19], [20]. One such adhesin, curli (C), along with the production of cellulose, has been shown to enhance bacterial adherence necessary for formation of biofilms [21], [22]. C are thin aggregative fimbriae and act as a virulence factor by promoting attachment to eukaryotic cells [23], [24]. C fimbriae, encoded by *csg*A, are expressed in response to low temperature, low oxygen, low osmolarity, and nutrient limitation [25].

Subpopulations of EcO157 have been reported to adapt to harsh environmental conditions [26]. Mutations that result in the C^−^ phenotype may confer a selective advantage in surviving austere environments. C^−^ strains survive up to 10,000 times better than C^+^ strains under acidic conditions (*p*H 2.4) and this appears to occur by maintaining C^−^ cells regardless of selection pressure [27]. Thus, it is possible that some environmental conditions cause selection of subpopulations with enhanced fitness to contaminate produce and, amplify enough to cause significant illness and an outbreak.

We described differences in predation of EcO157 by different protozoa isolated from dairy wastewater in a previous study [14]. In this study, we evaluate predation by *V. microstoma* and *C. aspera* of clinical and environmental strains of EcO157 that are highly related genotypically and associated with the 2006 spinach outbreak. In addition, we compared if specific phenotype subpopulations that evade protozoan predation are increased in fitness for survival in soil from produce field.

## Materials and Methods

### Ethics statement

Soil samples were provided by the Western Center for Food Safety and Security, University of California at Davis. No special permits were required as the soils were collected under cooperative agreements with produce growers and the samplings did not involve any endangered or protected species.

### Strains of EcO157 used in this study

EcO157 strains (Table 1) were selected based on sample source and genetic similarities determined by multi-locus variable-number tandem-repeat analysis (MLVA) and reported in a previous study [28]. Clinical and environmental strains of four different MLVA types (Table S1) associated with the 2006 spinach outbreak were used in these comparisons. All but one of the strains was highly related by 11-loci MLVA; strain RM9834 differed from the others at 9 loci. All of them carry virulence genes, *stx*2 (Shiga-toxin), *eae* (intimin), and *hly* (hemolysin); and serotype specific genes, *fli*C (H7-antigen) and *rfb*E (O157-antigen) [28]–[30]. Purity of cultures was confirmed by plating on Rainbow agar (Biolog, Hayward, CA) containing novobiocin (20 µg/ml, Sigma-Aldrich) and tellurite (0.8 µg/ml, Invitrogen/Dynal) (Rainbow-NT).

**Table 1:** EcO157 strains associated with 2006 spinach outbreak.

Strain No.	MLVA type	Source	State	Details^a^
RM6441	176	Cow feces	CA	CDPH-FDLB, Paicines ranch
RM6103	163	Cow feces	CA	CDPH-MDL, Paicines ranch
RM6088	176	Cow feces	CA	CDPH-MDL, Paicines ranch
RM6096	163	Cow feces	CA	CDPH-MDL, Paicines ranch
RM6440	176	Cow feces	CA	CDPH-FDLB, Paicines ranch
RM6157	176	Feral pig feces	CA	Paicines ranch
RM6106	174	Feral pig feces	CA	Paicines ranch
RM6155^c^	163	Feral pig feces	CA	Paicines ranch
RM9834^c^	778	Soil	CA	CSREES Environmental Study, Ranch J^b^
RM9993	163	Spinach bag	PA	CDC
RM6067^c^	163	Spinach bag	PA	Pennsylvania Department of Health
RM6068	163	Spinach bag	PA	Pennsylvania Department of Health
RM9996	163	Spinach bag	PA	CDC
RM6331^c^	163	Clinical	OR	Oregon State Public Health Lab
RM6653^c^	163	Clinical	WI	CDC
RM6069^c^	163	Clinical	PA	Pennsylvania Department of Health
RM6654^c^	163	Clinical	NM	CDC
RM6657^c^	163	Clinical	UT	CDC

a CDPH-FDLB, California Department of Public Health – Food and Drug Laboratory Branch; CDPH-MDL, California Department of Public Health – Microbial Diseases Laboratory; CSREES, USDA Cooperative state Research, Education, and Extension Service; and CDC, Centers for Disease Control.

b Isolated repeatedly from pasture soil during a 45-day period during 2009. All other strains were isolated during the outbreak period.

c Strains used in the soil fitness study.

### Preparation of inoculums and enumeration of EcO57

Isolated colonies from Rainbow-NT agar were grown over-night in 10% Luria broth (LB, Fisher Scientific, PA) at 25°C and at 150 rpm on a gyratory shaker, cells were separated by centrifuging at 10,000×*g* for 5 min and washed twice in 0.01 M PBS (*p*H 7.0) and adjusted to OD_600_ of 0.3 prior to inoculations to Sonneborn medium (Solution 1 of ATCC medium 802, http://www.atcc.org/Attachments/4018.pdf) or soil. Enumeration of EcO157 from soils or protozoa media was carried out by plating 100 µl volumes of 10-fold serial dilutions in 0.01 M PBS onto Rainbow-NT agar and the bluish-grey colonies were counted after over-night incubation at 37°C. Some colonies were tested at random by real-time PCR to confirm the presence of O157-antigen specific gene *rfb*E, using a method described previously [28].

### Enumeration of phenotypic variant subpopulations

The proportion of phenotypic variants of EcO157 strains, post-protozoan or soil exposure, expressing C was determined as described previously [27], with some modifications. Briefly, twenty isolated colonies from each of three replicates, confirmed as EcO157 on Rainbow-NT agar plates initially, were patched on LB agar without NaCl, but supplemented with 40 µg/ml of Congo red dye and 10 µg/ml of Coomassie brilliant blue G (Congo red agar). Thus, a total of 60 colonies for each strain of EcO157 were analyzed for determining the proportion of C variants. C^+^ (red) and C^−^ (white) colonies were tested at random to confirm the presence of *rfb*E [28]. The proportion of C subpopulations prior to soil or protozoan exposure were determined by plating serial dilutions of over-night growth from LB agar onto Congo red agar.

### Consumption of EcO157 strains by protozoa

Consumption of EcO157 strains was determined using *V. microstoma* and *C. aspera* isolated previously [14] from dairy wastewater. Twenty-five milliliters of sterilized 10% Sonneborn medium in 0.01 M PBS (*p*H 7.0) supplemented with 5% 3-µm filtered and heat-killed wastewater was inoculated with 1×10^8^ CFU/ml of EcO157 and 2×10^3^ cells of protozoa per ml (50,000 bacteria/protozoa). A 7-day old growth of protozoa in Sonneborn medium was used as inoculum. Overnight growth of EcO157 strains in 10% LB broth, centrifuged and resuspended in 0.01 M PBS was used as the bacterial food source for protozoa. The populations of both EcO157 and protozoa during a 7-day incubation without agitation at 25°C were determined using methods described previously [14] except that 5-fold serial dilutions in 0.01 M PBS were used for counting protozoa by the MPN method. The comparisons were in triplicate and days for 90% decreases (D-value) of EcO157 as a result of consumption by protozoa were calculated. Stationary incubations aid in grazing of EcO157 by micro-vortexing and filter feeding by the ciliates. Two-way ANOVA coupled with Bonferroni post t-test (Prism 4.0; GraphPad Software, Inc., San Diego, CA) or Holm-Sidak pairwise multiple comparisons (Sigmaplot v11, Systat software, Inc., Chicago, IL) were used to compare differences in growth of protozoa and differential uptake of EcO157 by protozoa. Changes in C subpopulations as influenced by protozoa also were statistically analyzed.

### Survival of EcO157 in soil from a produce field

Survival of nine EcO157 strains (Table 1) was monitored in the <45 µm fraction of fine soil (US standard sieve, 325 mesh, Hogentogler, Columbia, MD) collected from a produce field from lower Salinas Valley in Monterey County, CA (Farm R). Soil samples from this field were cultured by methods described previously for the presence of EcO157 [28]. One gram samples of soil in 4-ml screw capped glass vials (Wheaton Science Products, Millville, NJ) were inoculated with ∼1×10^7^ CFU of EcO157 cells in 160 µl and adjusted to moisture at 50% water holding capacity (26.1% moisture on dry weight basis) by adding 110 µl sterile distilled water. Vials containing the inoculated soils were capped loosely to allow aeration, incubated at 25°C and sampled at regular intervals for the enumeration of surviving EcO157 cells and for analyzing the proportion of C subpopulations as described above. The moisture loss at each sampling interval was monitored by weighing another set of vials containing un-inoculated soil. Soils were mixed thoroughly with sterile spatulas and ∼100 mg soil samples by weight were removed and used to make 10-fold serial dilutions for enumeration of EcO157. D-values were calculated based on the decreases in EcO157 populations during a 7-day incubation period. All the comparisons were in triplicates and the data was analyzed statistically as described above. In addition, Spearman rank order correlations were used to compare the fitness of EcO157 in soil with resistance to predation; growth increases of protozoa and the proportion of C subpopulations.

## Results

### Strain differences in uptake and utilization of EcO157 for protozoan growth

**Figure 1:**
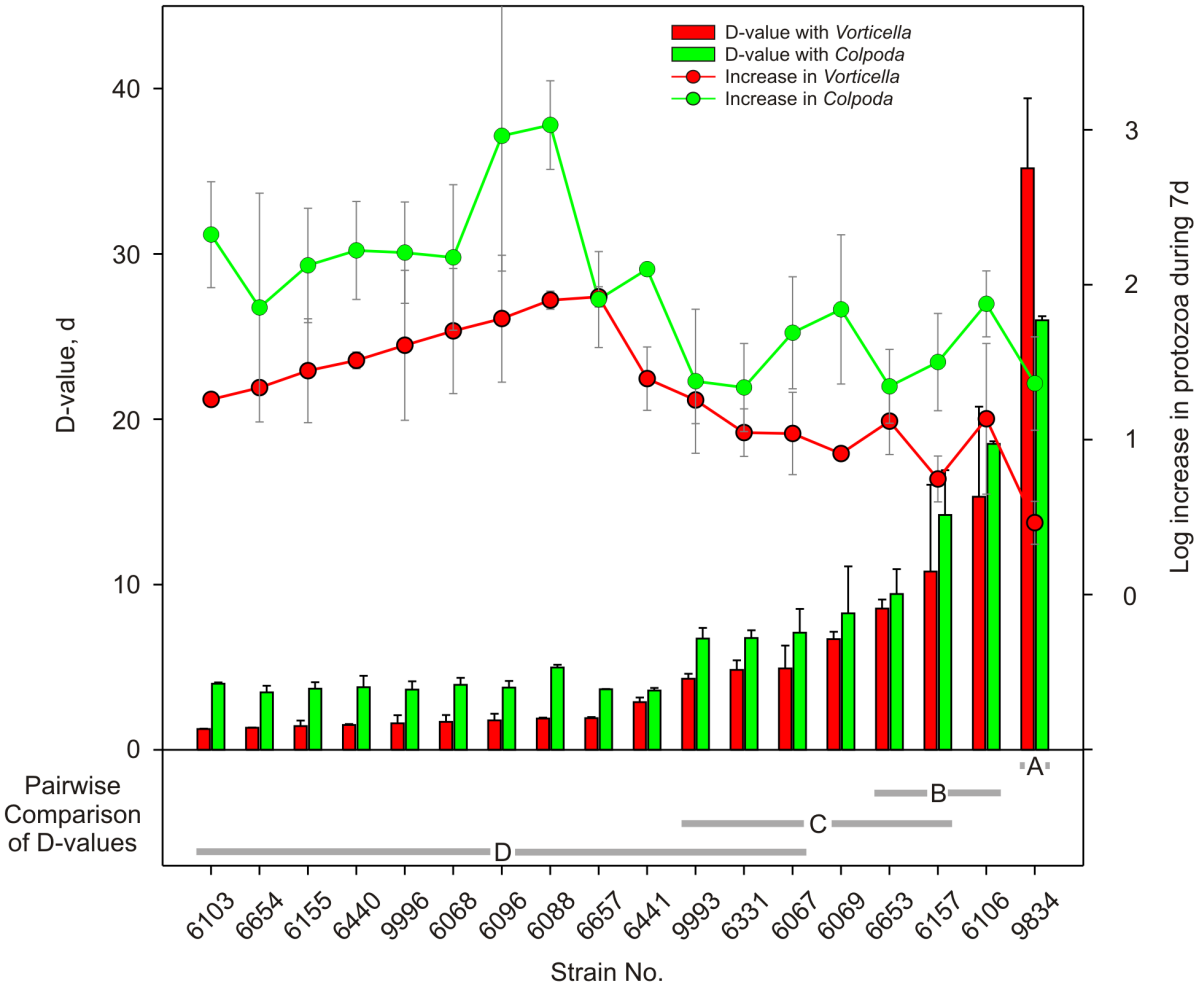
Inter-strain differences in preferential predation of EcO157 by protozoa from dairy wastewater. Survival of EcO157 populations measured as D-values (bar graph) and resultant increases in log numbers (line graph) of *V. microstoma* and *C. aspera* are shown.

Environmental and clinical strains of EcO157 associated with the 2006 spinach outbreak (Table 1) were fed to *V. microstoma* and *C. aspera* to determine if any strains resist predation or, alternatively, were consumed preferentially based on their genetic differences or source of isolation. Inter-strain differences in predation of EcO157 isolates were indicated by a wide range of D-values from 1 to 35 days in the presence of *Vorticella* and 4 to 26 days for *Colpoda* (Figure 1). Strain RM9834, isolated from pasture soil (Table 1), resisted predation by both *Colpoda* and *Vorticella*, as indicated by longer D-values (26 to 35 d) (Figure 1). All strains of EcO157 grew slightly (0.5 to 1 log increase) in the absence of predation during the 7-day incubation and thus D-values for EcO157 without protozoa are not shown.

Both protozoa grew by consuming EcO157; the log increases in protozoa correlated inversely with D-values (Figure 1, Table 2). Log increases of protozoan numbers were significantly higher (*P*<0.001; Table S2) with *Colpoda* compared to *Vorticella* (Figure 1). Inherent strain differences and their sources of isolation influenced protozoan growth significantly (*P*<0.001) resulting from the consumption of EcO157 cells (Table S2). An isolate from soil, two from feral pig feces (RM6106, RM 6157) and two clinical isolates (RM6653, RM6069) resisted protozoan predation compared to strains isolated from cow feces and bagged spinach.

**Table 2:** Influence of C-variant proportions on the survival of EcO157 strains before or after exposure to protozoa and in soil from a spinach field.

Variables correlated	Correlation coefficient, *r*_S_ a	*P*-value	Significance^c^
**C proportion prior to exposure** and			
D-values with soil^b^	−0.683	0.036	*
D-values with *Colpoda*	−0.467	0.049	*
D-values with *Vorticella*	−0.129	0.603	NS
Growth increase of *Colpoda*	0.130	0.597	NS
Growth increase of *Vorticella*	0.450	0.060	NS
**C proportion with ** ***Colpoda*** and			
D-values with *Colpoda*	−0.750	0.0001	****
Growth increase of *Colpoda*	0.625	0.006	**
**C proportion with ** ***Vorticella*** and			
D-values with *Vorticella*	−0.465	0.05	*
Growth increase of *Vorticella*	0.730	0.0004	***
**D-values** **of EcO157 with ** ***Colpoda*** and			
Growth increase of *Colpoda*	−0.554	0.017	*
**D-values** **of EcO157 with ** ***Vorticella*** and			
Growth increase of *Vorticella*	−0.674	0.002	**
**D-values of EcO157 in soil** b and			
D-values with *Colpoda*	0.067	0.844	NS
D-values with *Vorticella*	−0.100	0.775	NS

a Spearman rank-order correlations.

b Fate of EcO157 in soil compared with 9 strains of different proportions of C (see Table 3 for proportion of C variants prior to exposure to soil).

c *  =  *P*<0.05, **  =  *P*<0.01, ***  =  *P*<0.001, ****  =  *P*<0.0001 and NS  =  not significant.

Tests with strains grouped based on MLVA typing (Table S1) indicated that genetic differences influenced significantly (*P*<0.001, Table S2) the consumption of EcO157 by protozoa (Figure 1). Single strains representing MLVA types 778 and 174 resisted predation and survived significantly longer (*P*<0.001) than the clinical outbreak strains of MLVA 163. These results indicate that strains that are highly related by MLVA can have functional fitness differences depending upon the environment to which it was exposed.

### Protozoan exposure alters the proportion of C variant subpopulations of EcO157

**Figure 2:**
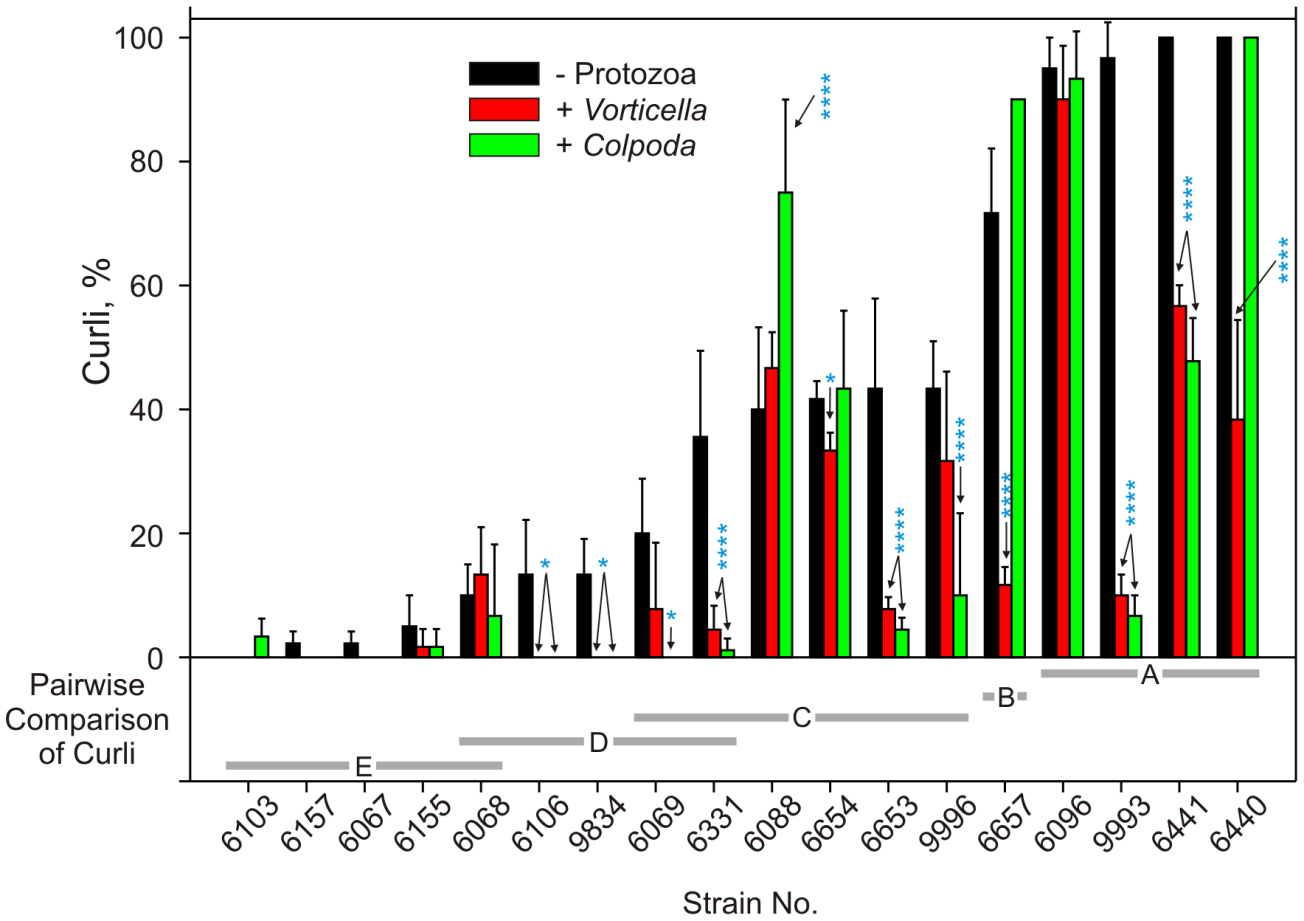
Effect of protozoan predation on curli-variant subpopulations of EcO157 strains. Proportion of C variants was monitored both in the presence or absence of protozoa. Values are averages of triplicates. Bonferroni post tests: *  =  *P*<0.05; **  =  *P*<0.01; ***  =  *P*<0.001; ****  =  *P*<0.0001.

Strains with significant differences (*P*<0.001, Table S2; Figure 2) in proportion of C variants were evaluated to check if protozoa consume subpopulations of EcO157 preferentially. The proportion of C^+^ subpopulations decreased significantly (*P* = 0.006) during the 7-day incubation with both protozoa and even more significantly with strains RM6441, RM9993 and RM6331 and RM6553 (*P*<0.0001). However, C^−^ strains (RM6103, RM6157, RM6155, RM6067; Figure 2) after the protozoan exposure remained C^−^ and were consumed with comparatively low growth increases (Figure 1). In addition, significant decreases in the proportion of C^+^ variants occurred specifically with some strains in co-culture with *Vorticella* and with different strains with *Colpoda* (Figure 2, Table S2,). C^+^ variants of RM6440 and RM6657 exposed to *Vorticella* decreased very significantly compared to *Colpoda* (*P*<0.0001). The proportion of C^+^ variants in the presence of protozoa was correlated negatively with D-values (*P*<0.05; Table 3) of EcO157 and correlated positively with log increases of protozoa (*P*<0.01). Thus, log increases in protozoa correlated negatively with D-values of EcO157 strains (*P*<0.05; Table 3, Figure 1).

**Table 3:** EcO157 survival in soil and proportion of curli sub-populations of parental strains.

Strain No^a^	Source	D-value, days	Curli, %
RM6103	Cow feces	10.0±1.2	0±0
RM6155	Pig feces	12.3±0.6	5±5
RM6067	Spinach	10.3±2.5	2±2
RM9834	Soil	10.8±2.4	13±6
RM6069	Clinical	4.0±2.6	20±9
RM6331	Clinical	7.2±0.5	36±14
RM6653	Clinical	5.7±1.3	43±15
RM6654	Clinical	6.0±1.6	42±3
RM6657	Clinical	5.5±0.8	72±10

a Except for RM9834 (MLVA 778) all other strains are of MLVA 163.

### Strain differences in soil fitness and protozoan predation

The fate of EcO157 in soil was monitored to evaluate if strains that resist predation also survive longer in soil. Nine strains from fitness studies with protozoa (Table 1) were used in these comparisons; eight are genetically indistinguishable by MLVA typing from the major MLVA type of the spinach outbreak strain (MLVA 163). A pasture soil isolate, RM9834 (MLVA 778), was included to compare the soil fitness of strains highly related by MLVA, but from different sources, to an unrelated strain. Environmental strains survived longer in soil (high D-values) compared to clinical isolates (Table 3), but no correlation was apparent between soil persistence and resistance of EcO157 for predation by protozoa (Table 2). Strain differences were associated with significant variance in D-values (*P*<0.0001; Table 3). However, the soil isolate, RM9834, survived longer in soil and also resisted predation significantly (Figure 1; Table 3). Similar to the predation resistance discussed above (Table 2 and S2), persistence of EcO157 in soil was also correlated negatively (*r*_s_ = −0.683; *P* = 0.036) with the proportion of C variants. Only C^−^ variants remained in soil after a 7-day exposure of 7 out of 9 strains. The proportions of C^+^ and C^−^ variants of the other two strains (RM6657, RM6069) were not evaluated.

## Discussion

EcO157 is prevalent in agricultural environments (such as dairies, feedlots) and has been detected frequently in environmental samples during and after the 2006 spinach outbreak [2], [28]. The pathogen has been reported to persist in manure piles for nearly 2 years [31]. Conversely, some strains (including a strain linked to apple juice outbreak) disappeared from dairy wastewater in less than a day [16]. These results indicate that EcO157 strains respond differently to different environmental and biological factors. Indeed, one of the strains we used in this study, RM9834, was isolated repeatedly from a naturally contaminated dry pasture soil during a 45-day period indicating that at least some cells remained viable under harsh environmental stress. Similarly, bacterial pathogens that can avoid predation by protozoa and the capability of growing under low nutrient conditions and/or inhibitory chemicals in wastewater [15] have a higher probability of surviving under stressful conditions of produce production environments.

EcO157 strains from environmental sources such as soil and feral pig feces, in particular, resisted predation by both protozoa significantly (Figure 1, Table S2). These strains were co-isolated along with the outbreak strain from feral pigs and cow feces [2] during the 2006 spinach outbreak or, subsequently, from pasture soil. The soil isolate (RM9934) differed phylogenetically from the outbreak strains; tandem repeats at 9 out of 11 loci were different (Table S1). Environmental stresses can be associated with subtle changes in the MLVA tandem repeats resulting in increased phylogenetic diversity [32], but it is not known if environmental exposures may improve the chances of survival and resistance to predation of pathogenic EcO157.

Both protozoa we tested consumed strains from clinical samples, spinach and cow feces; *Vorticella* consumed EcO157 cells more rapidly (Figure 1, shorter D-values; *P* = 0.031) compared to *Colpoda* (Table S2). Consumption of *E. coli* and EcO157 in preference to native bacteria, in soils [33] and dairy wastewater [14], respectively, has been reported, but inter-strain differences in consumption of EcO157 by environmental protozoa has not been described. In addition, we show that outbreak strains from clinical and environmental samples were consumed rapidly, compared to environmental strains from pig feces and soil, isolated from the same vicinity (Figure 1). These results indicate that protozoa may be a significant factor in eliminating many EcO157 soon after their release into dairy lagoons through feces.

Protozoa grew as they consumed EcO157 strains rapidly and, as expected, protozoan growth was correlated negatively with D-values of EcO157 (Figure 1, Table 3). However, *Colpoda* grew significantly (Figure 1, Table S2) compared to *Vorticella*, although *Vorticella* eliminated EcO157 more rapidly. To our knowledge, there have been no reports related to the growth of environmental protozoa after consumption of strains of EcO157. Significant increases in growth of both protozoa were observed for nearly half-of the strains (Figure 1) that are highly related genetically to the outbreak strain. In contrast, both protozoa grew significantly less (*P*<0.001) with isolates that resisted predation and were from feral pig feces and soil. Similarly, protozoa that can feed and grow on EcO157 when released into dairy ponds might explain why EcO157 were not isolated in a previous study from dairy wastewater [16].

In addition to genetic differences of EcO157 strains and their prior exposure to environmental stresses that influence their relative resistance to predation, intra-strain differences in C expression also influenced predation by protozoa. A high proportion of C^+^ to C^−^ variants correlated negatively with D-values indicating significant consumption of EcO157 resulting in growth increases of both protozoa (Table 3). Thus, strains from cow feces, spinach bags and some clinical isolates with a high proportion of C^+^ variants were rapidly consumed by both protozoa and decreased the proportion of C^+^ variants very significantly (*P*<0.0001). In contrast, strains from feral pig feces and dry pasture soil were predominantly C^−^ (Figure 2) and resisted predation. Although no other data on predation resistance of subpopulations has been reported, a C-deficient EcO157 strain was reported to survive better on plant surfaces [34], presumably, because plant defense systems recognize C and influence the survival of subpopulations. Alternatively, some bacteria with hydrophobic cell surfaces resist predation [35] making it worth determining if similar mechanisms are responsible for predation resistance of C^−^ strains or subpopulations.

We hypothesize that EcO157 strains with increased percentage of C^−^ variants have a fitness mechanism to survive under hostile produce production soil environments with extremes in moisture and temperature conditions. Thus, we isolated RM9834 with 87% C^−^ subpopulations from dry pasture soil. Furthermore, a 7-day exposure of 3 clinical and 4 environmental strains (one of each from cow feces, feral pig feces, soil and bagged spinach) in spinach field soil resulted in recovering only C^−^ subpopulations. This previous environmental exposure is probably associated with extended survival in soil when it is re-inoculated into soil, and may be due to C^−^ strains being isolated predominately from these environments; this is consistent with the proportion of C^+^ variant subpopulations correlating negatively (*r*_S_ = −0.683, *P*<0.05) with soil D-values (Table 2). We speculate that naturally C^−^ environmental isolates can survive well in the moderately acidic soil used in this study (*p*H 5.4), and is consistent with significant acid stress-resistance reported for C^−^ variants of EcO157 strains [27]. Acid stress-resistance is critical for EcO157 surviving the acidic conditions in the stomach of ruminants and humans, and perhaps other environments, and it is intriguing that, generally, environmental strains survive acid stress much better than the isolated clinical or food strains associated with outbreaks [36]. Similarly, all clinical strains we analyzed decreased in numbers more rapidly from soil compared to environmental strains.

The fitness responses observed for survival of EcO157 under stressful conditions in soil and resisting predation by protozoa were quite similar. Both environments elicited a similar phenotypic response of C^−^ variants, predominately, surviving the exposure, a negative correlation of proportion of C^+^ variants with D-values of EcO157 and a positive correlation with growth of both predators. Since 8 of the 9 strains used in these tests are genetically indistinguishable from the 2006 spinach outbreak strain by MLVA (163), soil fitness or predation resistance of these isolates cannot be attributed to genetic differences. However, the naturally persistent, but genetically different, soil isolate (MLVA778), which was 87% C^−^ variants, resisted predation by both protozoa and also survived longer in spinach field soil. Thus, it appears that most pathogenic EcO157 fail to survive in hostile soil and water environments, but selection and enrichment of C^−^ variants is one survival strategy that may relate to contamination of produce.

In summary, these results indicate that the environmental persistence of pathogenic EcO157 is associated with the fitness of clonal subpopulations, although some generalizations can be made on survival in soil and resistance to predation based on isolation source. Whereas populations of clinical strains associated with the spinach outbreak decreased rapidly in soils or were consumed rapidly by protozoa, environmental strains (from soil and feral pig feces) survived longer in soil and resisted predation. Our results suggest also that exposure of EcO157 strains to stress conditions (e.g. soil or protozoa) generally result in rapid die-off, but the subpopulations that do survive are usually C^−^. Thus, the presence of C^−^ variants in a population of cells at some frequency appears to be a survival mechanism associated with EcO157 fitness in soil and on plant surfaces [34], resisting protozoan predation, and potentially, other environmental stresses or conditions to be discovered. The mechanisms of C variation (e.g. *rpoS*) and the role of C^+^ and C^−^ variants in complex environments warrant further studies to determine whether selection of some variants are associated also with hypervirulent strains associated with outbreaks.

## Supporting Information

Table S1 MLVA characteristics of EcO157 strains used. Number of tandem repeats at each of 11 loci are given.
(DOCX)

Table S2 Two-way ANOVA comparisons of strain differences in fitness of EcO157 to protozoan predation, protozoan growth response to EcO157 consumption and influence of protozoan exposure on proportion of curli subpopulations.
(DOCX)
