# Supplementary material for: Strain Differences in Fitness of Escherichia coli O157:H7 to Resist Protozoan Predation and Survival in Soil
Source: PLoS One. 2014 Jul 14;9(7):e102412. doi: 10.1371/journal.pone.0102412 (PMC4097067; doi:10.1371/journal.pone.0102412)
Supplement: Table S1 — MLVA characteristics of EcO157 strains used. Number of tandem repeats at each of 11 loci are given. (DOCX) [file pone.0102412.s001.docx]

**Table S1.** MLVA characteristics of EcO157 strains used. Number of tandem repeats at each of 11 loci are given.

| Variable number of tandem repeats at each locus | | | | | | | | | | | | |
| --- | --- | --- | --- | --- | --- | --- | --- | --- | --- | --- | --- | --- |
| MLVA | Strains tested^b^ | Vhec1 | Vhec2 | Vhec3 | Vhec4 | Vhec5 | Vhec6 | Vhec7 | O157-17 | O157-19 | O157-25 | O157-37 |
| 163^a^ | 6103, 6096, 6155, 9993, 6067, 6068, 9996, 6331, 6653, 6096, 6654, 6657 | 16 | 9 | 11 | 17 | 7 | 9 | 9 | 4 | 7 | 5 | 6 |
| 778 | 9834 | 35 | 9 | 17 | 13 | - | 9 | 13 | 6 | 8 | 4 | 11 |
| 174 | 6106 | 14 | 9 | 11 | 16 | 7 | 9 | 9 | 4 | 7 | 5 | 5 |
| 176 | 6088, 6440, 6441, 6157 | 14 | 9 | 11 | 17 | 7 | 9 | 9 | 4 | 7 | 5 | 6 |

^a^ Strains in this group are also indistinguishable by their PFGE patterns

^b^ The prefix ‘RM’ from each strain number is omitted for brevity.
