# Supplementary material for: Strain Differences in Fitness of Escherichia coli O157:H7 to Resist Protozoan Predation and Survival in Soil
Source: PLoS One. 2014 Jul 14;9(7):e102412. doi: 10.1371/journal.pone.0102412 (PMC4097067; doi:10.1371/journal.pone.0102412)
Supplement: Table S2 — Two-way ANOVA comparisons of strain differences in fitness of EcO157 to protozoan predation, protozoan growth response to EcO157 consumption and influence of protozoan exposure on proportion of curli subpopulations. (DOCX) [file pone.0102412.s002.docx]

Table S2. Two-way ANOVA comparisons of strain differences in fitness of EcO157 to protozoan predation, protozoan growth response to EcO157 consumption and influence of protozoan exposure on proportion of curli subpopulations.

| Dependent variable | Independent variable | *F*-statistic | *P*-value | Significance of differences between group means^a^ |
| --- | --- | --- | --- | --- |
|  |  |  |  |  |
| D-values^b^ | Protozoa | 0.06 | 0.807 | *Colpoda* ^NS^ *Vorticella* |
|  | MLVA | 55.4 | <0.001 | 778***>174***>176=163 |
|  |  |  |  |  |
| D-values | Protozoa | 5.5 | 0.031 | *Colpoda* *>*Vorticella* |
|  | EcO157 strains | 26.7 | <0.001 | A 9834  B 6106  BC 6157  BCD 6653  CD 6069, 6067, 6331, 9993  D 10 others |
|  |  |  |  |  |
| D-values | Protozoa | <0.001 | 0.978 | *Colpoda* ^NS^ *Vorticella* |
|  |  |  |  |  |
|  | EcO157 isolation source | 30.7 | <0.001 | A Soil  B Feral pig feces  C Clinical, spinach bag, cow feces |
|  |  |  |  |  |
| Log increase in protozoa | Protozoa^b^ | 10.0 | 0.004 | *Colpoda* *>*Vorticella* |
|  | MLVA^b^ | 2.2 | 0.110 | All 4 MLVA groups^NS^ |
|  |  |  |  |  |
| Log increase in protozoa^b^ | Protozoa | 24.0 | <0.001 | *Colpoda* ***>*Vorticella* |
|  | EcO157 isolation source | 6.5 | <0.001 | A Cow feces  AB Spinach bag  BC Feral pig feces, clinical, soil |
|  | Source within *Vorticella* |  |  | All 4 sources^NS^ |
|  | Source within *Colpoda* |  |  | A Cow feces  AB Spinach bag  ABC Feral pig feces  BC Clinical, soil |
|  | Strains within each isolation source | 1.3 | 0.274 | NS |
|  |  |  |  |  |
| Log increase in protozoa^b^ | Protozoa | 66.4 | <0.001 | *Colpoda* ***>*Vorticella* |
|  | EcO157 strains | 6.2 | <0.001 | A 6088  AB 6096, 6068  ABC 6657, 9996, 6440, 6103, 6155, 6441, 6654, 6106  BC 6069, 6067, 9993  C 6653, 6331, 6157, 9834 |
|  |  |  |  |  |
|  | Protozoa within each strain^c^ | 95.2 | <0.0001 | *Colpoda* *>*Vorticella*: 6088, 6096, 6103, 6069, 9834 |
|  |  |  |  |  |
| Proportion of C variants | EcO157 strains^b^ | 7.6 | <0.001 | A 6096  AB 6440  ABC 6441, 6657, 6088, 6654, 9993  BC 9996  C 6653, 6331, 6069, 6068, 6106, 9834, 6155,6103, 6157, 6067 |
|  | Strains within both protozoa^c^ | 157.5 | <0.0001 | -Protozoa****>6441, 9993, 6331, 6653 |
|  | Strains within *Vorticella* |  |  | -Protozoa****>6440, 6657 |
|  | Strains within *Colpoda* |  |  | -Protozoa****>6088, 9996  -Protozoa *>6069 |
|  |  |  |  |  |
| Proportion of C variants^b^ | Protozoa^b^ | 5.9 | 0.006 | -Protozoa *> *Vorticella*^NS^ *Colpoda* |
|  | Protozoa within strains^c^ | 121.3 | <0.0001 | *Colpoda*****>*Vorticella*: 6440, 6657  *Colpoda* ***>*Vorticella*: 6088  *Vorticella* *>*Colpoda*: 9996 |
|  |  |  |  |  |

^a^ Groups arranged in the order of decreasing means preceded by the same letter designation are not significantly different from each other. A broken line demarcates a separate ANOVA using data groups as indicated. The prefix ‘RM’ from each strain number omitted for brevity.

^b^ Two-way ANOVA with Holm-Sidak pairwise multiple comparison post-hoc t-tests.

^c^ Two-way ANOVA with Bonferroni multiple comparisons.
